# Supplementary material for: Treadmilling FtsZ polymers drive the directional movement of sPG-synthesis enzymes via a Brownian ratchet mechanism
Source: Nat Commun. 2021 Jan 27;12:609. doi: 10.1038/s41467-020-20873-y (PMC7840769; doi:10.1038/s41467-020-20873-y)
Supplement: Supplementary file 1 — Supplementary Information [file 41467_2020_20873_MOESM1_ESM.pdf]

Supplementary Information

Treadmilling FtsZ polymers drive the directional movement of sPG-synthesis enzymes *via* a Brownian ratchet Mechanism

Joshua W. McCausland[1], Xinxing Yang[1], Georgia R. Squyres[2], Zhixin Lyu[1], Kevin E. Bruce[3], Melissa M. Lamanna[3], Bill Söderström[4], Ethan C. Garner[2], Malcolm E. Winkler[3], Jie Xiao[1*], and Jian Liu[5*]

1. Department of Biophysics and Biophysical Chemistry, Johns Hopkins School of Medicine, Baltimore, MD, 21205, USA;

2. Department of Molecular and Cellular Biology, Harvard University, Cambridge, MA, 02138, USA

3. Department of Biology, Indiana University Bloomington, Bloomington, IN, 47405 USA

4. The ithree institute, University of Technology Sydney, Ultimo, NSW, 2007 Australia

5. Department of Cell Biology, Johns Hopkins School of Medicine, Baltimore, MD, 21205, USA.

(*) Corresponding Authors: xiao@jhmi.edu and jliu187@jhmi.edu

This PDF includes:

Supplementary Tables
Supplementary Figures
Supplementary References

Supplementary Tables

	Source	Abundance (relative value to FtsZ in parentheses) FtsZ	FtsI/PBP2b/PBP2x	FtsW
E. coli	Arike et al.[1]	585 (1)	0.21 (0.00359)	0.14 (0.00239)
	Krug et al.[2]	1291 (1)	1.97 (0.00152)	2.12 (0.00164)
	PRIDE PRD000418[3]	507 (1)	13.8 (0.02722)	1.97 (0.00389)
	Valgepea et al.[4]	956 (1)	36.3 (0.03797)	27.4 (0.02866)
	Arike et al.[1]	942 (1)	59.0 (0.06263)	12.6 (0.01337)
	Lu et al.[5]	1376 (1)	11.5 (0.00836)	0.91 (0.00066)
	Lewis et al.[6]	887 (1)	3.83 (0.00432)	0.86 (0.00097)
	PRIDE PRD000485[7]	811 (1)	27.5 (0.03391)	6.01 (0.00741)
	PRIDE PRD000485[7]	721 (1)	NA	6.11 (0.00847)
	PAX db 4.1[8]	833 (1)	0.25 (0.00030)	NA
B. subtilis	Chi et al.[9]	744 (1)	87.5 (0.11761)	53.0 (0.07124)
	PAX db 4.1[8]	894 (1)	97.5 (0.10906)	19.9 (0.02225)
	PAX db 4.1[8]	909 (1)	98.5 (0.10836)	16.6 (0.018262)
S. pneumoniae	Noirclerc-Savoye et al.[10]	NA	260	NA
	Lara et al.[11]	3000	NA	NA
	E. coli average relative abundance		0.01962	0.00726
	B. subtilis average relative abundance		0.11168	0.03725
	S. pneumoniae relative abundance		0.087	NA

Supplementary Table 1. **Comparison of PG synthase abundance between *E. coli, B. subtilis*, and *S. pneumoniae***. *E. coli* and *B. subtilis* data were gathered from the protein abundance database[8]. For *E. coli*, we pulled all results from searching "FtsI," "FtsW," or "FtsZ." For *B. subtilis* we pulled all results from searching "PBPb", "FtsW," or "FtsZ." *S. pneumoniae* estimates were obtained from the literature[10,11].

Position	Mutation	Annotation	Gene	Description
257,908	Δ776bp		*insB9–[crl]*	*insB9, insA9, [crl]*
361,267	Δ6303bp		*[lacA]–lacI*	*[lacA],lacY,lacZ,lacI*
1,978,503	Δ776bp		*insB-5-insA-5*	*insB-5, insA-5*
1,995,620	IS*5* (–) +4b	Intergenic (-261/-32)	*uvrY* ← *I* → yecU	DNA-binding transcriptional activator UvrY/protein YecU
2,173,363	Δ2 bp	Pseudogene (915-916/1358nt)	*gatC* ←	Galactitol-specific PTS enzyme IIC component
2,958,626	C → T	A87T (GCA → ACA)	*ptrA* ←	protease 3
3,560,455	+G	Pseudogene (151/758nt)	*glpR* ←	DNA-binding transcriptional repressor GlpR
4,296,381	+GC	Intergenic (+587/+55)	*glltP* → *I* ← *yjcO*	Glutamate/aspartate: H(+) symporter GltP/Sel1 repeat-containing protein YjcO

Supplementary Table 2. **Identified mutations and deletions in the parent strain TB28 from whole genome sequencing in comparison to *E. coli* strain MG1655**. JM136 shared these same perturbations in addition to insertion of HaloTag at FtsI's N-terminus.

Name	Purpose	Sequence
JM26	Amplifying pACYC vector (R)	GTGCACATTATACGAGCCGATGA
JM35	Amplifying sgRNA insert (R)	CGGCGTAGAGGATCCACAGGACGGGTGTGGTCGC
JM44	Amplifying pACYC vector (F)	ATCATGGCGACCACACCCGTCCTGTGGATCCTCTAC
JM97	Amplifying *ftsI*$^{18\text{-}19}$ sgRNA (F)	TCGTATAATGTGCACAACATGCCAACTTTATCAGTGTTTTAGAGCTAGAAATAGCAAGTTAA
JM95	Amplifying *halotag-ftsI*$^{18\text{-}19}$ repair oligo (F)	AAGCAGCGGCGAAAACGCAGAAACCAAAACGTCAGGAAGAACACGCAAACGGTGGCGGTGGTAGCGGATCCGAAATCGGTACTGGC
JM96	Amplifying *halotag-ftsI*$^{18\text{-}19}$ repair oligo (R)	AGCGCCAGGAGAATACAGCCGCATAACAACGCAAAACGCCATGAAATGAAGCTACCACCGCCACCGGAAATCTCCAGAGTAGAC

Supplementary Table 3. **Primers used in this study**. JM97 contains a 20nt protospacer for amplifying the sgRNA. JM95 and JM96 have GGGGS linkers encoded in their sequence in addition to the 50nt overhangs with the genomic site for λ-Red insertion.

Strain	Genotype	Reference/Source
TB28	*E. coli* MG1655 *lacIYZ* <> *frt*	Bernhardt 2003[12]
JM136	TB28 ftsI::halotag-ftsI$^{18\text{-}19}$	This Study
EC182	*E. coli* MG1655 *ftsI*::*cat* (*attL-lom*)::*bla lacIQ pBad-ftsI*	Wissel & Weiss 2004[13]
bGS28	*B. subtilis* PY79 *pbp2B::erm-P*$_{hyperspank}$*-HaloTag-15aa-pbp2B*	Bisson-Filho 2017[14]
IU15096	*S. pneumoniae* D39 Δ*cps rpsL1 ftsW*-L_0-*ht*-P_C-*erm*	Perez 2019[15]
**Plasmid**	**Genotype**	**Reference/Source**
pXY018	pCA24N, *lacIQ*, *GFP-ZapA*	Yang 2017[16]
pRM027	$P_{t5\text{-}lac}$::*meos3.2-ftsI*	This Study
pKD46	repA101(ts), ParaB$^-$ (gam bet exo)	Datsenki 2000[17]
pGH33	pET28a, P_{bad}::*spCas9*, *ampR*	This Study
pGH34	pACYC, *cmR*, P_{bad}::*sacB*, P_{bad}::*sgRNA*	This Study
pJM44	pACYC, *cmR*, P_{bad}::*sacB*, P_{bad}::*ftsI*$^{18\text{-}19}$*sgRNA*	This Study

Supplementary Table 4. **Strains and plasmids used in this study.**

Supplementary Figures

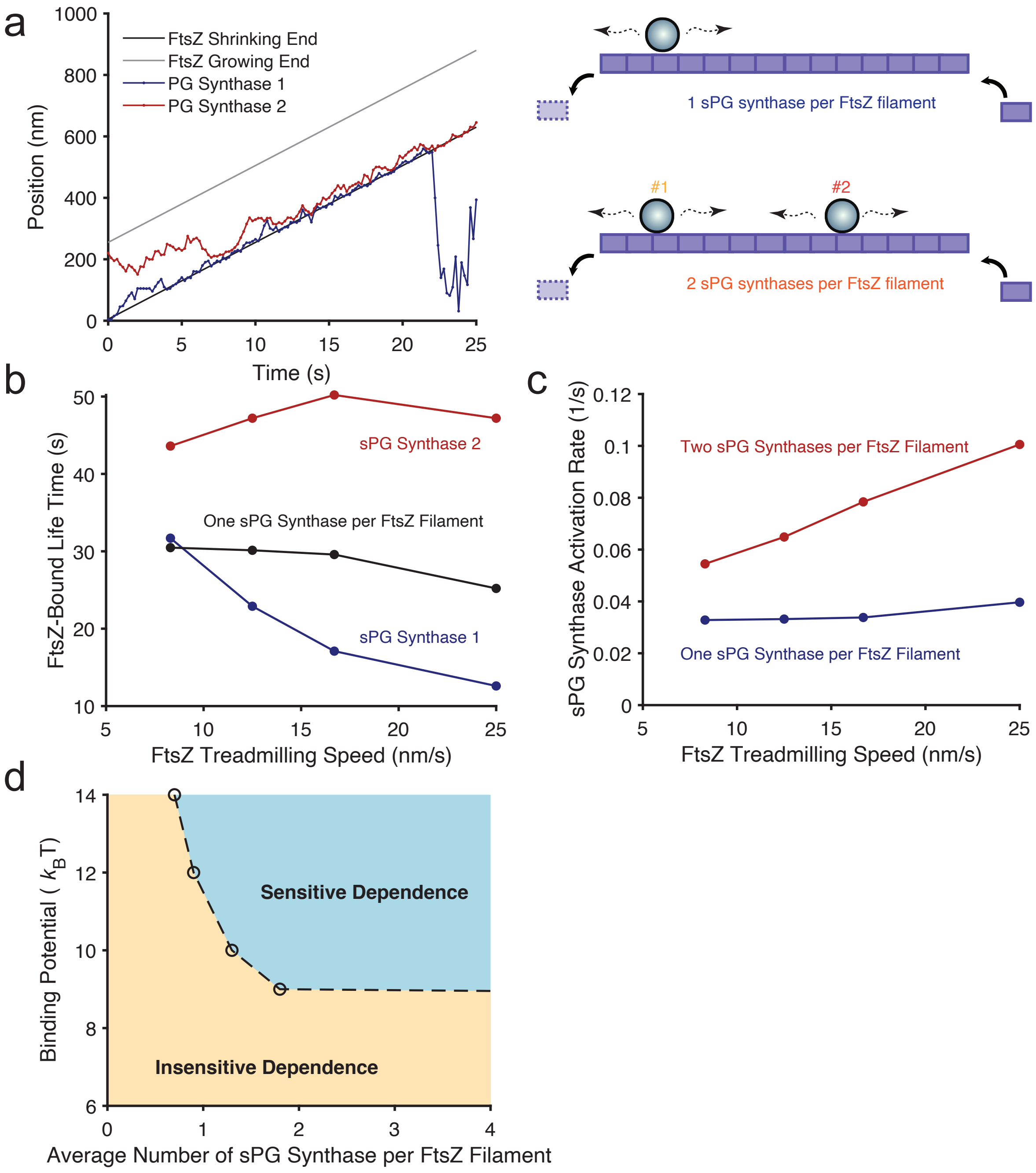

Supplementary Figure 1. **Abundance of sPG synthase modulates FtsZ treadmilling speed-dependence of cell wall synthesis. a** Representative simulation trajectory of two sPG synthases on a treadmilling FtsZ filament. *Left*: Schematic of two sPG synthases on the same treadmilling FtsZ filament, as compared to the case of one sPG synthase. Note that as the two sPG synthases move along the one-dimensional track of a FtsZ filament,

one of them will start to end-track the FtsZ shrinking end, with the hops around in the middle of the FtsZ filament. As the first pass, the only interaction between the sPG synthases considered by the current model is a short-ranged volume exclusion preventing them from crossing each other – when the two complexes touch each other, they “bounce” apart by 5 nm. As such, when the two sPG synthases approach each other, the end-tracking one will be “knocked” off the track and the other will take over the baton and start end-tracking. **b** Dependence of FtsZ-bound sPG synthase lifetime on FtsZ treadmilling speed. **c** sPG synthase levels modulate the dependence of sPG synthase activation rate (i.e., the inverse of the lifetime in **b**) on FtsZ treadmilling speed. **d** sPG synthase level and FtsZ-binding potential govern the sensitivity of sPG synthase activation on FtsZ treadmilling speed. With the FtsZ treadmilling speed increasing 3-fold (from 8.3 to 25 nm/s), we counted it as “insensitive-dependence”, if the corresponding changes in sPG synthase activation rate is less than 30%, relative to the activation rate at FtsZ treadmilling speed of 8.3 nm/s. Otherwise, we counted it as “sensitive-dependence”. For (**a** – **d**), 100 independent stochastic simulation trajectories were simulated and averaged for each data point, wherever it applies. If not otherwise mentioned, the nominal parameter set is that the diffusion constant of free sPG synthases is 0.04 $\mu m^2/s$, sPG synthase-FtsZ binding potential 10 $k_B T$, and FtsZ treadmilling speed 25 nm/s.

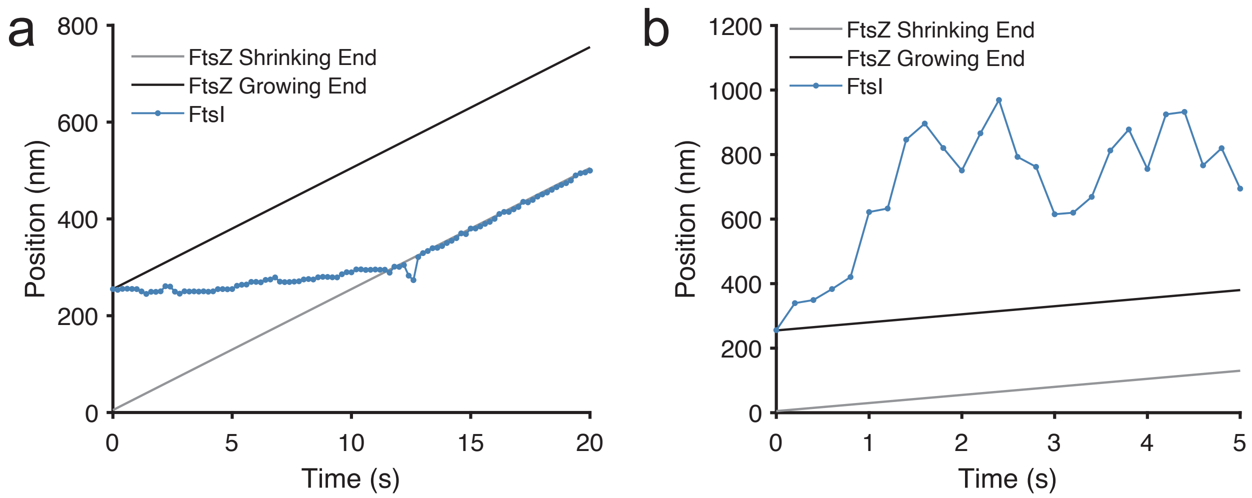

Supplementary Figure 2. **Model results of FtsI movement at the FtsZ growing end. a** A representative simulated trajectory of a slow-diffusing FtsI. It gets stuck at the FtsZ growing end until it can persistently end-track the FtsZ shrinking end. **b** A representative simulated trajectory of a fast-diffusing FtsI. It escapes from the FtsZ growing end before the FtsZ shrinking end approaches it. The diffusion constant of free FtsI is set to be 0.01 mm^2/s in A) and 0.04 mm^2/s in **b**. The rest of the parameters are the same for **a** and **b**: FtsI-FtsZ binding potential is 10 kT and FtsZ treadmilling speed is 25 nm/s.

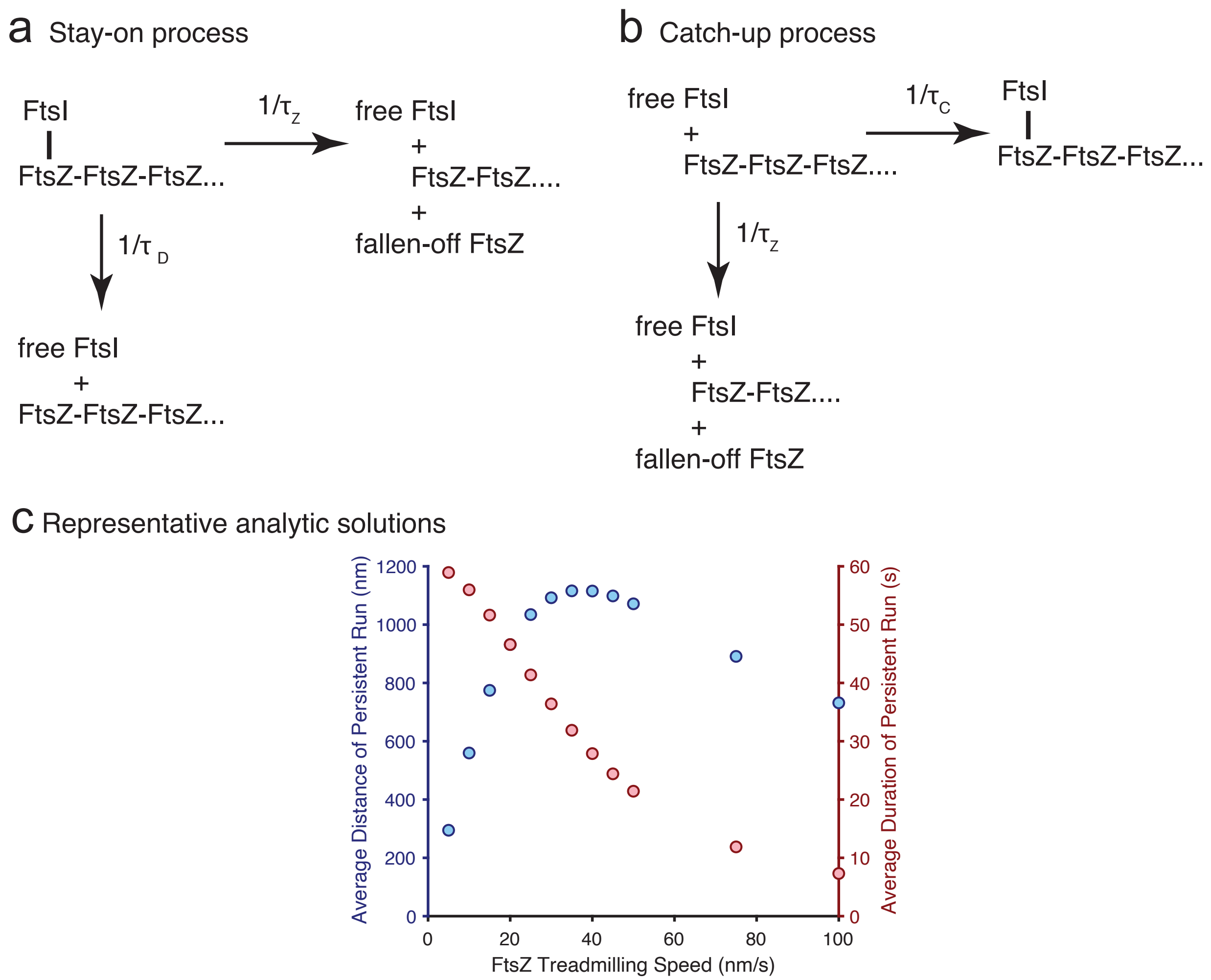

Supplementary Figure 3. **Model results of analytic solution.** **a** Schematic of chemical reactions for the FtsI bound to the FtsZ end subunit. **b** Schematic of chemical reactions for the free FtsI to catch up with the FtsZ end subunit. **c** FtsZ treadmilling speed-dependence of the average run distance and duration of FtsI persistent end-tracking. Here, the plot is calculated based on the analytic solutions of Supplementary Equations [3-4], where $\tau_D = 60\ s$ and $\tau_C = 0.0003\ s$. As long as $\tau_D >> \tau_Z >> \tau_C$, the essence of the plot holds up.

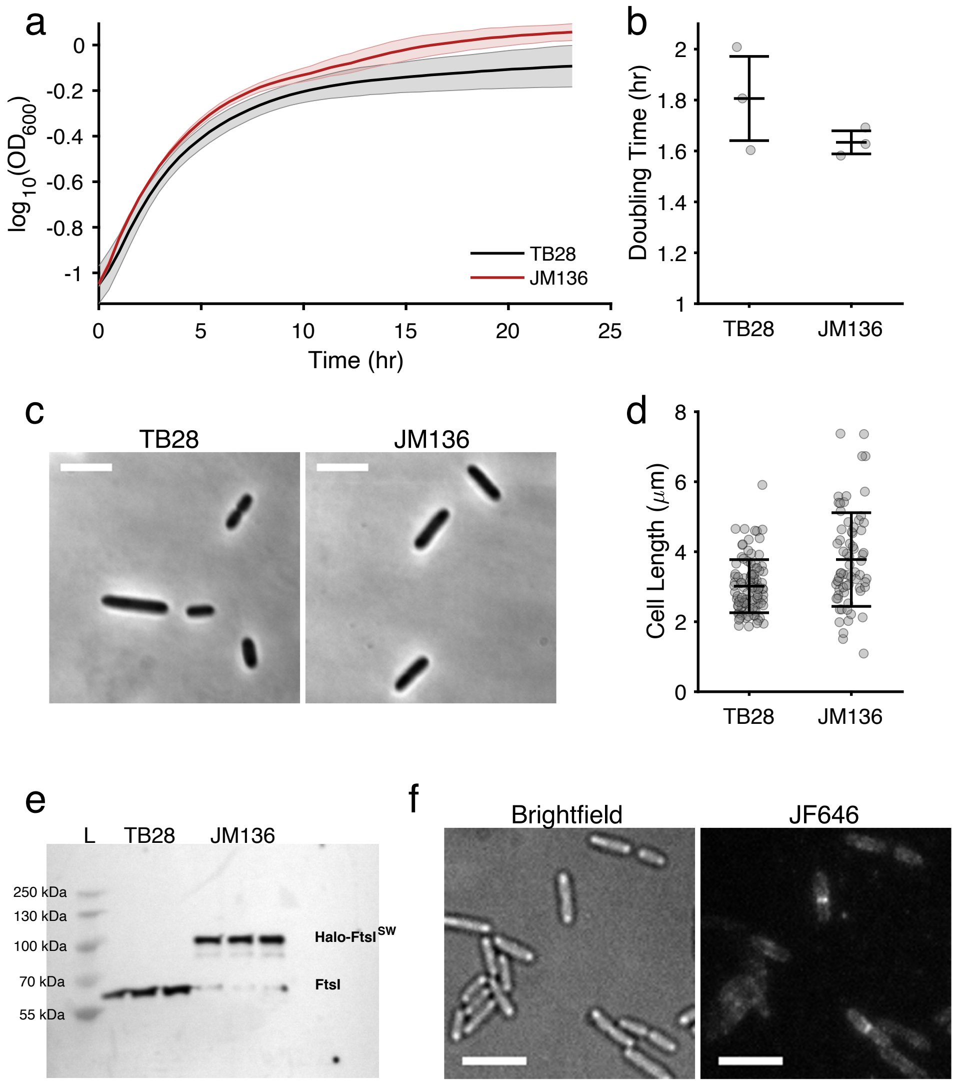

Supplementary Figure 4. **Characterization of JM136. a** Growth curve of TB28 vs. JM136 (with Halo-FtsI) in M9 minimal media at 30 °C (mean ± S.E.M., n = 3 biological replicates). **b** TB28 vs. JM136 doubling time, calculated from the growth curve in **a** (mean ± standard deviation, n = 3 biological replicates). **c** Phase contrast images of TB28 and JM136. Scale bar is 5µm. Experiment repeated three times with similar results. **d** Cell length of TB28 vs. JM136, measured from Oufti[18] from phase contrast images (mean ± standard deviation, n = 1 biological replicate. TB28 n = 98 cells, JM136 n = 68 cells). **e** Western blot of FtsI in TB28 and JM136 (three biological replicates each, experiment was repeated 2 other times with similar results). "L" denotes the lane the ladder

was run in. **f** Halo-FtsI localizes mid-cell in Halo-FtsI. Cells were stained with 1μM JF646. Scale bar is 5μm. Experiment was repeated three times with similar results

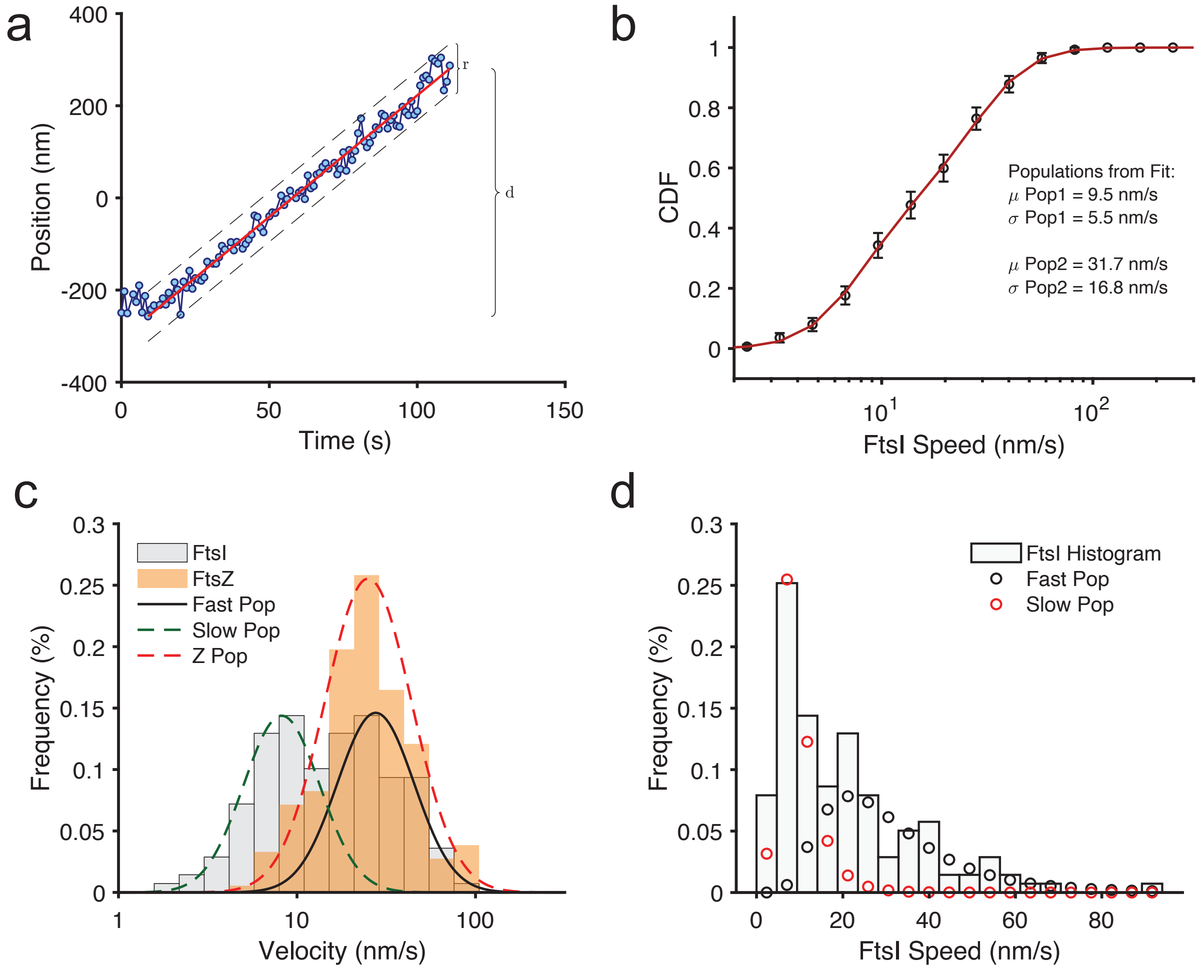

Supplementary Figure 5. **Segmentation and deconvolution of FtsI directional segments. a** To determine the noise-to-signal ratio $R = \frac{r}{d}$, we use the residual and displacement information from fitted segments. **b** A two-population CDF fit to the raw velocity data (139 processive segments in n = 49 cells, 18 biological replicates). Parameters were measured on the ln-scale, where P = 0.49 ± 0.15, μ_1 = 2.1 ± 0.14, σ_1 = 0.50 ± 0.15, μ_2 = 3.3 ± 0.20, σ_2 = 0.50 ± 0.12 (all mean ± S.E.M, derived from bootstrapping the data n = 200 times). **c** Fitting the two FtsI populations to their separate fits, with FtsZ from Yang et al.[16] for comparison. **d** Raw FtsI histogram with the two-population overlay, used for deconvolution of the fast population.

Supplementary References

1 Arike, L. *et al.* Comparison and applications of label-free absolute proteome quantification methods on Escherichia coli. *Journal of Proteomics* **75**, 5437-5448 (2012).

2 Krug, K. *et al.* Deep coverage of the Escherichia coli proteome enables the assessment of false discovery rates in simple proteogenomic experiments. *Molecular and Cellular Proteomics* **12**, 3420—3430, doi:10.1074/mcp.M113.029165 (2013).

3 Huang, S.-T. (Proteomics Identification Database, 2011).

4 Valgepea, K. *et al.* Systems biology approach reveals that overflow metabolism of acetate in Escherichia coli is triggered by carbon catabolite repression of acetyl-CoA synthetase. *BMC Systems Biology* **4**, 1-13, doi:https://doi.org/10.1186/1752-0509-4-166 (2010).

5 Lu, P., Vogel, C., Wang, R., Yao, X. & Marcotte, E. M. Absolute protein expression profiling estimates the relative contributions of transcriptional and translational regulation. *Nature Biotechnology* **25**, 117-124, doi:https://doi.org/10.1038/nbt1270 (2007).

6 Lewis, N. E. *et al.* Omic data from evolved E. coli are consistent with computed optimal growth from genome-scale models. *Molecular Sytems Biology* **6**, 1-13, doi:10.1038/msb.2010.47 (2010).

7 (ed Attila Csordas) (Proteomics Identification Database, 2012).

8 Wang, M., Herrmann, C. J., Simonovic, M., Szklarczyk, D. & Mering, C. v. Version 4.0 of PaxDb: Protein abundance data, integrated across model organisms, tissues, and cell lines. *Proteomics* **15**, 3163—3168, doi:10.1002/pmic.201400441 (2015).

9 Chi, B. K. *et al.* S-Bacillithiolation protects against hypochlorite stresss in Bacillus subtilis as revealed by transcriptomics and redox proteomics. *Molecular and Cellular Proteomics* **10**, 1-21, doi:10.1074/mcp.M111.009506 (2011).

10 Noirclerc-Savoye, M. *et al.* In vitro reconstitution of a trimeric complex of DivIB, DivIC, and FtsL, and their transient co-localization at the division site in Streptococcus pneumoniae. *Molecular Microbiology* **55**, 413—424 (2005).

11 Lara, B. *et al.* Cell division in cocci: localization and properties of the Streptococcus pneumoniae FtsA protein. *Molecular Microbiology* **55**, 699-711 (2005).

12 Bernhardt, T. G. & de Boer, P. A. J. The Escherichia coli amidase AmiC is a periplasmic septal ring component exported via the twin-arginine transport pathway. *Molecular Microbiology* **48**, 1171-1182 (2003).

13 Wissel, M. C. & Weiss, D. S. Genetic analysis of the cell division protein FtsI (PBP3): Amino acid substitutions that impair septal localization of FtsI and recruitment of FtsN. *Journal of Bacteriology* **186**, 490—502, doi:10.1128/JB.186.2.490–502.2004 (2004).

14 Bisson-Filho, A. W. *et al.* Treadmilling by FtsZ filaments drives peptidoglycan synthesis and bacterial cell division. *Science* **355**, 739-743, doi:10.1126/science.aak9973 (2017).

15 Perez, A. J. *et al.* Movement dynamics of divisome proteins and PBP2x:FtsW in cells of Streptococcus pneumoniae. *Proceedings of the National Academy of Sciences* **116**, 3211-3220, doi:10.1073/pnas.1816018116 (2019).

16 Yang, X. *et al.* GTPase activity–coupled treadmilling of the bacterial tubulin FtsZ organizes septal cell wall synthesis. *Science* **355**, 744-747, doi:10.1126/science.aak9995 (2017).

17 Datsenko, K. A. & Wanner, B. L. One-step inactivation of chromosomal genes in Escherichia coli K-12 using PCR products. *Proceedings of the National Academy of Sciences* **97**, 6640-6645, doi:10.1073/pnas.120163297 (2000).

18 Paintdakhi, A. *et al.* Oufti: an integrated software package for high-accuracy, high-throughput quantitative microscopy analysis. *Molecular Microbiology* **99**, 767-777, doi:10.1111/mmi.13264 (2016).
