# Supplementary material for: Treadmilling FtsZ polymers drive the directional movement of sPG-synthesis enzymes via a Brownian ratchet mechanism
Source: Nat Commun. 2021 Jan 27;12:609. doi: 10.1038/s41467-020-20873-y (PMC7840769; doi:10.1038/s41467-020-20873-y)
Supplement: Supplementary file 3 — Reporting Summary [file 41467_2020_20873_MOESM3_ESM.pdf]

nature research

Corresponding author(s): Jie Xiao
Jian Liu

Last updated by author(s): Dec 4, 2020

Reporting Summary

Nature Research wishes to improve the reproducibility of the work that we publish. This form provides structure for consistency and transparency in reporting. For further information on Nature Research policies, see our Editorial Policies and the Editorial Policy Checklist.

Statistics

For all statistical analyses, confirm that the following items are present in the figure legend, table legend, main text, or Methods section.

n/a	Confirmed	
☐	☒	The exact sample size (*n*) for each experimental group/condition, given as a discrete number and unit of measurement
☐	☒	A statement on whether measurements were taken from distinct samples or whether the same sample was measured repeatedly
☐	☒	The statistical test(s) used AND whether they are one- or two-sided<br>*Only common tests should be described solely by name; describe more complex techniques in the Methods section.*
☒	☐	A description of all covariates tested
☒	☐	A description of any assumptions or corrections, such as tests of normality and adjustment for multiple comparisons
☐	☒	A full description of the statistical parameters including central tendency (e.g. means) or other basic estimates (e.g. regression coefficient) AND variation (e.g. standard deviation) or associated estimates of uncertainty (e.g. confidence intervals)
☒	☐	For null hypothesis testing, the test statistic (e.g. F, t, r) with confidence intervals, effect sizes, degrees of freedom and P value noted<br>*Give P values as exact values whenever suitable.*
☒	☐	For Bayesian analysis, information on the choice of priors and Markov chain Monte Carlo settings
☒	☐	For hierarchical and complex designs, identification of the appropriate level for tests and full reporting of outcomes
☒	☐	Estimates of effect sizes (e.g. Cohen's *d*, Pearson's *r*), indicating how they were calculated

Our web collection on statistics for biologists contains articles on many of the points above.

Software and code

Policy information about availability of computer code

Data collection	Custom journals were written in Metamorph 7.8.13.0 software to acquire time-stream single-molecule images. Computational analyses were completed in C.
Data analysis	ImageJ software and the ThunderSTORM plug-in were used to identify fluorescent spots for single-molecule tracking analysis. Data were processed with custom programming codes written in Matlab for spots linking and velocity analysis. See Methods section of manuscript for details. Codes are uploaded to the Xiao Lab's GitHub and cited with DOI in references.

For manuscripts utilizing custom algorithms or software that are central to the research but not yet described in published literature, software must be made available to editors and reviewers. We strongly encourage code deposition in a community repository (e.g. GitHub). See the Nature Research guidelines for submitting code & software for further information.

Data

Policy information about availability of data

All manuscripts must include a data availability statement. This statement should provide the following information, where applicable:
- Accession codes, unique identifiers, or web links for publicly available datasets
- A list of figures that have associated raw data
- A description of any restrictions on data availability

Source data for Figures 2-5 and Supplementary Figures 1-5 are provided with the paper as a Source Data file. The authors declare that all other data supporting the findings of this study are available within the paper and its supplementary information files, or from the corresponding authors upon request.

Field-specific reporting

Please select the one below that is the best fit for your research. If you are not sure, read the appropriate sections before making your selection.

- [x] Life sciences
- [ ] Behavioural & social sciences
- [ ] Ecological, evolutionary & environmental sciences

For a reference copy of the document with all sections, see nature.com/documents/nr-reporting-summary-flat.pdf

Life sciences study design

All studies must disclose on these points even when the disclosure is negative.

Sample size	For growth curve measurements, three biological replicates were completed.<br>For immunoblot, seven biological repeats were completed across three repeat experiments.<br>For single molecule tracking experiments, 77 trajectories (from 18 biological replicates and 49 cells) were collected to construct the histogram and scatterplots.<br>5000 trajectories were collected to calculate the E. coli MSD.<br>6765 trajectories were collected to calculate the B. subtilis MSD.<br>25 trajectories were collected to calculate the S. pneumoniae MSD.<br>Except for representative trajectories, each sample in computational work was based on 20 runs.
Data exclusions	Fluorescence spots with an intensity over 3 fold of the mean intensity were removed from analysis since they likely include more than one molecule. Short trajectories (shorter than 5 time points) of a single molecule are excluded because they cannot be accurately quantified or classified as moving or diffusive molecules.
Replication	Each biological repeat in our experiments is based on different source colonies of E. coli from the same genotype. The imaging experiments were completed at different days with other conditions such as temperature or cell density kept as similar as possible.
Randomization	By the way we repeat our experiments, the data are intrinsically randomized and independent.
Blinding	For single molecule tracking, the data collection is intrinsically blinded since the information from single molecules was randomly collected and not able to be discretely chosen with bias. Data analysis was not blinded considering the analysis pipeline is more computer-based.

Reporting for specific materials, systems and methods

We require information from authors about some types of materials, experimental systems and methods used in many studies. Here, indicate whether each material, system or method listed is relevant to your study. If you are not sure if a list item applies to your research, read the appropriate section before selecting a response.

Materials & experimental systems

n/a	Involved in the study	
☐	☒	Antibodies
☒	☐	Eukaryotic cell lines
☒	☐	Palaeontology and archaeology
☒	☐	Animals and other organisms
☒	☐	Human research participants
☒	☐	Clinical data
☒	☐	Dual use research of concern

Methods

n/a	Involved in the study	
☒	☐	ChIP-seq
☒	☐	Flow cytometry
☒	☐	MRI-based neuroimaging

Antibodies

Antibodies used	Anti-FtsI serum (From Dr. David S. Weiss)
Validation	Anti-FtsI has been verified, published, and noted in our Materials and Methods.
